# Laparoscopic Repair of an Incarcerated Parahiatal Hernia with Gastric Necrosis: A Rare Case Report

**DOI:** 10.70352/scrj.cr.25-0281

**Published:** 2026-06-03

**Authors:** Kazuyoshi Mitta, Toshikatsu Tsuji, Renta Kobori, Haruka Kubo, Shunsuke Takenaka, Hiroyuki Tanaka, Ryosuke Machi, Hiroshi Saito, Kenta Doden, Yusuke Sakimura, Hiroto Saito, Daisuke Yamamoto, Hideki Moriyama, Jun Kinoshita, Noriyuki Inaki

**Affiliations:** Department of Gastrointestinal Surgery, Kanazawa University Hospital, Kanazawa, Ishikawa, Japan

**Keywords:** parahiatal hernia, gastric necrosis, laparoscopic surgery, diaphragmatic hernia, hernia incarceration

## Abstract

**INTRODUCTION:**

Primary parahiatal hernias are rare in adults. We report the case of a young woman with a parahiatal hernia without obvious predisposing factors that resulted in gastric fundus necrosis.

**CASE PRESENTATION:**

A 34-year-old woman had experienced intermittent chest pain for 2 months but had not sought medical attention. She developed sudden-onset left chest pain without a history of trauma, with the pain worsening on inspiration. Her previous physician suspected a left lung abscess and referred her to our facility. Contrast-enhanced CT revealed a cavity with internal air above the diaphragm in the left thoracic cavity, which was continuous with the stomach in the abdominal cavity. We diagnosed an incarcerated diaphragmatic hernia with gastric ischemia and proceeded with emergency laparoscopic surgery. Intraoperatively, the gastric fundus was found to be incarcerated in the left diaphragm, and the hernial orifice was located just to the left of the esophageal hiatus. A diagnosis of parahiatal hernia was confirmed. The incarcerated gastric fundus was repositioned into the abdominal cavity. The hernial orifice was closed using nonabsorbable barbed sutures, and the necrotic gastric fundus was resected. The postoperative course was uneventful, and the patient was discharged on POD 13. CT at the 1-month follow-up revealed no recurrence of the parahiatal hernia.

**CONCLUSIONS:**

Parahiatal hernia is extremely rare but may be life-threatening if diagnosed late. In this case, laparoscopic repair and resection of the necrotic gastric fundus were successfully performed.

## Abbreviations

CRP
: C-reactive protein
ICG
: indocyanine green
WBC
: white blood cell

## INTRODUCTION

Adult diaphragmatic hernias, excluding esophageal and hiatal hernias, are rare. Among these, parahiatal hernias are even more uncommon.^1)^ We describe the case of a young woman with a parahiatal hernia without any apparent predisposing factors that resulted in gastric fundus necrosis.

## CASE PRESENTATION

**Fig. 1:**
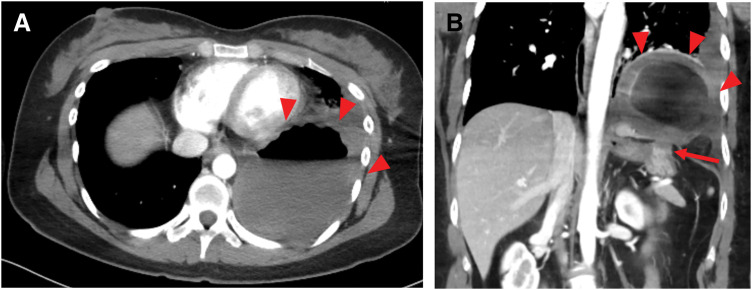
Representative images of contrast-enhanced CT at the first visit. (**A**) Axial image. The arrowheads indicate the incarcerated gastric fundus, which was visualized as a cavitary lesion with an air–fluid level in the thoracic cavity. (**B**) Coronal image. The arrowheads indicate the incarcerated gastric fundus; the arrow indicates the hernial orifice. The incarcerated fundus was visualized as a cavitary lesion in the thoracic cavity and was continuous with the stomach in the abdominal cavity at the hernial orifice. It exhibited poor contrast.

**Fig. 2:**
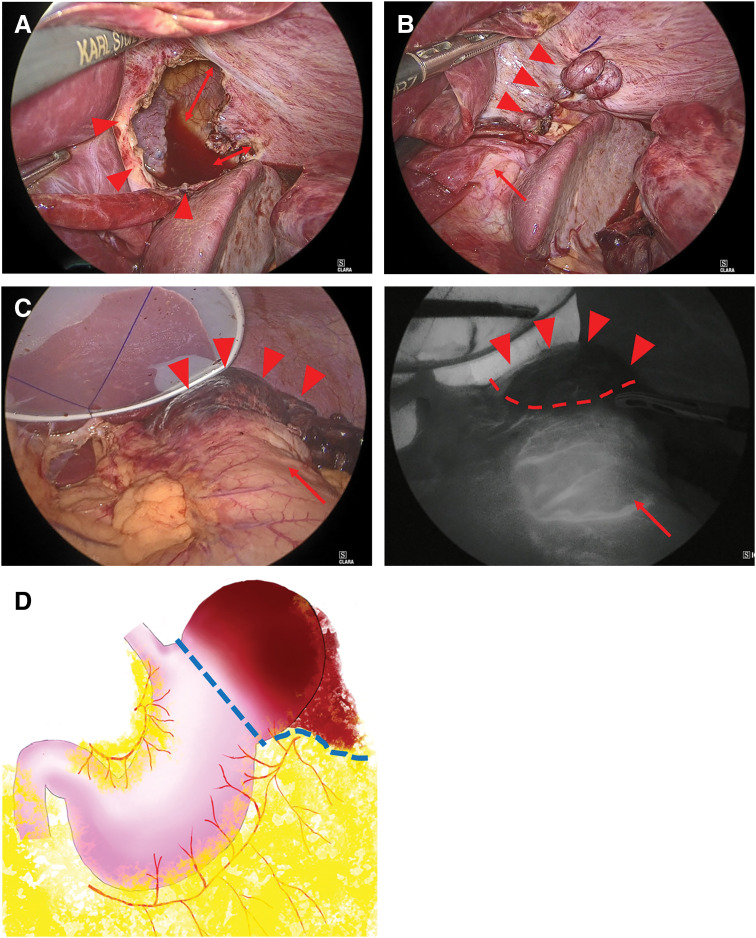
Intraoperative photograph. (**A**) Intraoperative photograph showing the hernial orifice. The arrowheads indicate the hernial orifice after it was incised and dilated. The arrow shows the extent of the incisions made from the hernial orifice. (**B**) Photograph of the closed hernial orifice. The arrowheads indicate closure of the hernial orifice using a barbed suture. The arrow shows the esophageal hiatus. The hernial orifice is located ventrally and to the left side of the esophageal hiatus. (**C**) Photographs of the necrotic gastric fundus. The left image shows the appearance under white light, with the arrowheads marking the necrotic area. The right image shows ICG fluorescence imaging, which revealed an absence of fluorescence in the gastric fundus. The red dashed line indicate the demarcation lines for resection. (**D**) Schematic illustration showing the resection range of the stomach and greater omentum. The blue dashed line indicates the resection line.

**Fig. 3:**
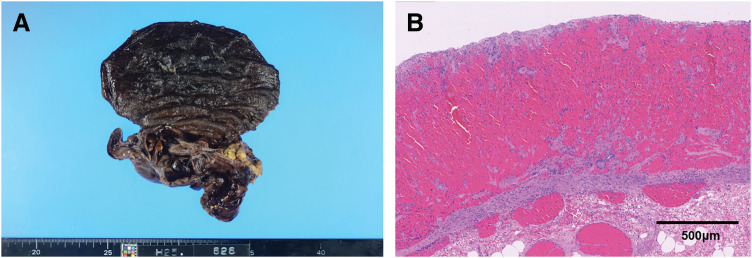
Resected gastric fundus. (**A**) Macroscopic image of the resected gastric fundus. (**B**) Microscopic image (hematoxylin and eosin staining). Transmural hemorrhage and necrosis were observed.

**Fig. 4:**
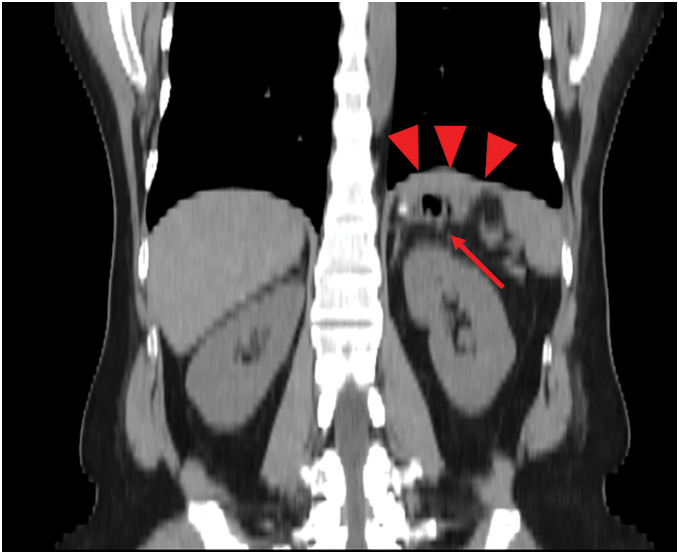
Representative image of postoperative CT. Postoperative CT image at the 1-month follow-up. The arrowheads show the diaphragm; the arrow shows the stomach. No recurrence of the parahiatal hernia was seen.

A 34-year-old woman with chronic polyarthritis, treated in our rheumatology department with prednisolone (2 mg/day) and methotrexate (10 mg/week), presented with sudden left-sided chest pain that worsened with inspiration. Although she had intermittently experienced chest pain for 2 months, she had not visited the hospital, and there was no history of trauma. She had never experienced any chest symptoms prior to the episode and had no abnormal shadows in any previous examinations. When she sought care at another hospital, a plain CT scan of the thorax and abdomen revealed a cavitary lesion in the left lower thoracic cavity, initially suggesting a lung abscess. She was referred to our hospital for further examination and treatment. Laboratory tests revealed a WBC count of 17.65 × 10^3^/μL and a CRP level of 3.55 mg/dL, indicative of systemic inflammation, as well as a lactate level of 2.5 mmol/L, raising concerns about possible organ ischemia. Contrast-enhanced CT of the thorax and abdomen revealed a cavitary lesion containing internal air in the left thoracic cavity above the diaphragm (**Fig. 1A** and **1B**). The lesion was continuous with the stomach, and the surrounding fatty tissue resembled the greater omentum, suggesting a diaphragmatic hernia in which portions of the stomach and greater omentum had prolapsed into the left thoracic cavity. Since the herniated organ appeared to have compromised blood flow, emergency surgery was performed. A laparoscopic exploration confirmed incarceration of the gastric fundus in the left diaphragm. The defect was located adjacent to the esophageal hiatus, consistent with parahiatal hernia (**Fig. 2A**). After partially incising and enlarging the hernial orifice, the incarcerated fundus was returned to the abdominal cavity. The hernia defect, measuring approximately 3 cm (excluding the incision), was then closed using nonabsorbable barbed sutures (**Fig. 2B**). Mesh placement was not performed because of the potential risk of mesh infection. The gastric fundus appeared dark red, suggesting necrosis; ICG fluorescence imaging was therefore used to assess the blood flow. A linear stapler was used to resect the necrotic portion of the fundus at the location where an adequate fluorescent signal was still present (**Fig. 2C** and **2D**). Pathological examination confirmed transmural hemorrhage and necrosis (**Fig. 3A** and **3B**). Postoperative recovery was uneventful, and the patient was discharged on POD 13. A follow-up CT performed 1 month later showed no recurrence of the parahiatal hernia (**Fig. 4**).

ICG, indocyanine green

## DISCUSSION

We report the first successful laparoscopic resection of a necrotic stomach segment caused by primary parahiatal hernia. Clinically similar conditions, including paraesophageal hernias or combined hernias, are subtypes of esophageal hiatal hernias, with several recent reports of incarceration.^2–5)^ However, unlike these cases, her esophageal hiatus was intact, confirming a parahiatal hernia. Cases requiring gastrectomy for gastric ischemia due to an incarcerated parahiatal hernia are exceedingly rare, with only 3 cases reported to date.^6–8)^ Moreover, all patients had secondary parahiatal hernias following surgery. In addition, this represents the first reported instance of ICG-guided gastric resection in a patient with parahiatal hernia. ICG-guided resection enabled the avoidance of excessive gastric loss and contributed to the preservation of the gastric cardia.

### Definition and classification of parahiatal hernia

Most diaphragmatic hernias detected in adulthood are esophageal hiatal hernias, whereas others are less common. Parahiatal hernia has an incidence of 0.2%–0.35%, as reported by Palanivelu et al.,^9)^ and is characterized by an intact esophageal hiatus and a hernial orifice near (most often to the left of) that hiatus.^10)^ Her hernial orifice was located on the left side of the esophageal hiatus. Intraoperative photographs showed that the hernial orifice appeared slightly more lateral owing to the leftward surgical incision, yet it was positioned closer to the central tendon of the diaphragm than typically observed in Bochdalek hernia, supporting the diagnosis of parahiatal hernia. Symptoms include cardiac discomfort, nausea, vomiting, postprandial chest pain that can mimic angina pectoris, early satiety, and respiratory complaints.^1)^ These hernias are classified as either primary or secondary. During early embryonic development, the thoracic and abdominal cavities communicate through the pleuroperitoneal canals. These canals normally close at approximately 7–8 weeks of gestation with the development of the pleuroperitoneal membranes, separating the thoracic and abdominal cavities. Primary parahiatal hernias stem from a congenital failure to close them. Secondary parahiatal hernias are independent of this and are triggered by factors such as age-related muscle weakness, increased intra-abdominal pressure, or iatrogenic factors (including injuries during hiatal hernia repair, junctional plasty, pleural neoplasm resection and esophagectomy).^10)^ We classified this as a primary parahiatal hernia because she was relatively young, had no history of trauma or surgery, and showed no predisposing factors.

### Review of previously reported cases

Using PubMed, we searched for the term “parahiatal hernia” and identified 38 cases reported in 21 articles.^6–26)^ The details are shown in **Table 1**. The primary type was more common than the secondary type (26 vs. 12), and female patients were more than male patients (27 vs. 11). Most of the parahiatal hernia cases have been treated laparoscopically (33 of 38 cases).

**Table 1:** Summary of previously reported parahiatal hernias

Authors	Cases	Age	Sex	Etiology	Surgical approach	Hernia content + condition	Organ resection	Mesh	Fundoplication
Vallieres and Waters^8)^	1	49	F	Secondary (transthoracic Heller myotomy)	Thoracotomy	Gastric fundus, perforated	Partially gastrectomy	No	No
Demmy et al.^12)^	1	48	F	Primary	Thoracotomy	Stomach, perforated, necrotic	No	No	No
Rodefeld and Soper^13)^	1	64	F	Primary	Laparoscopic	Gastric cardia and fundus	No	No	Nissen
Scheidler et al.^14)^	2	68	F	Primary	Laparoscopic	Gastric fundus and omentum	No	No	Nissen
		57	M	Primary	Laparoscopic	Gastric fundus	No	No	Nissen
Ganesh et al.^15)^	1	42	F	Primary	Laparoscopic	Fat and gastric fundus	No	No	No
Palanivelu et al.^9)^	8	Average 48.7 (range 29–70)	M: 5 F: 3	Primary: 4; secondary: 4 (laparoscopic transhiatal enucleation: 1; laparoscopic fundoplication: 3)	Laparoscopic: 8	Not reported: 8	No	Yes: 6; no: 2	Nissen: 2; no: 6
Ohtsuka et al.^11)^	1	39	M	Primary	Laparoscopic	Stomach and transverse colon	No	No	No
Takemura et al.^6)^	1	70	M	Secondary (malignant pleural mesothelioma resection)	Laparoscopic	Stomach, incarcerated	Proximal gastrectomy	No	No
Main and Tymitz^7)^	1	24	F	Secondary (robotic hiatal hernia repair and Nissen fundoplication)	Laparoscopic	Stomach, edematous and dusky	Longitudinal gastrectomy	Yes	No
Koh et al.^16)^	2	40	F	Primary	Laparoscopic	Stomach volvulus	No	No	No
		51	F	Primary	Laparoscopic	Stomach volvulus	No	Yes	Anterior 180°
Akiyama et al.^17)^	1	73	M	Secondary (thoracoscopic esophagectomy)	Laparoscopic	Transverse colon	No	Yes	No
Calin et al.^18)^	1	45	F	Primary	Robotic	Stomach	No	Yes	No
Staerkle et al.^19)^	1	71	M	Primary	Laparoscopic	Gastric fundus	No	Yes	Partial posterior
Preda et al.^20)^	1	60	F	Primary	Laparoscopic	40%–50% of the stomach	No	No	Nissen
Li et al.^21)^	2	73	F	Primary	Laparoscopic	Gastric fundus	No	Yes	No
		80	F	Primary	Laparoscopic	Gastric volvulus	No	Yes	No
Plourde and Comeau^22)^	1	62	F	Secondary (esophagectomy)	Laparotomy	Transverse colon and small bowel	Incarcerated transverse colon and small bowel resection	No	No
Kohama et al.^23)^	1	72	F	Primary	Thoracoscopic	Peritoneal mesothelioma	Peritoneal mesothelioma resection	No	No
Muramatsu et al.^24)^	1	39	F	Primary	Laparoscopic	Stomach	No	Yes	Toupet
Hany et al.^25)^	1	36	F	Secondary (laparoscopic sleeve gastrectomy)	Laparoscopic	Small intestine	No	Yes	No
Garcia and Chan^26)^	1	77	M	Primary	Laparoscopic	Omentum	No	No	Anterior Dor
		68	F	Primary	Laparoscopic	Stomach	No	No	Toupet
Han et al.^10)^	8	Average. 51.8 (range: 27–76)	M: 2 F: 6	Primary: 6; secondary: 2 (transthoracic esophageal repair for esophageal rupture: 1; multiple lumbar vertebrae surgeries: 1)	Laparoscopic: 8	Not reported: 8	No	Yes: 6; no: 2	180° anterior: 1; no: 8
Our case	1	34	F	Primary	Laparoscopic	Gastric fundus, incarcerated	Partially gastrectomy	No	No

F, female; M, male

The stomach is most frequently involved in parahiatal hernias; however, some cases involve the colon or small bowel. In adult Bochdalek hernia, the most frequent congenital diaphragmatic hernia, it was reported that the stomach was included in the hernia content in 40% of cases,^27)^ suggesting that the stomach is more frequently prolapsed in parahiatal hernias. Five cases requiring concomitant organ resection have been reported: 3 involving gastrectomy, 1 involving resection of the transverse colon and small intestine, and 1 involving resection of malignant peritoneal mesothelioma. Excluding the malignant peritoneal mesothelioma, 4 cases required resection because of ischemic necrosis. Among the 3 gastrectomy cases, 1 underwent partial gastrectomy, 1 longitudinal gastrectomy, and 1 proximal gastrectomy. Simple suture closure was performed in 16 cases, and mesh repair was performed in 22 cases. Fundoplication was performed in 11 cases, among which the Nissen procedure was the most commonly adopted technique (6 of 11 cases).

### Unique features of the present case

The present case is distinguished by several important features. One of the features is that the condition was initially misdiagnosed as a lung abscess. Another is that emergent laparoscopic resection was performed for the stomach incarcerated in a primary parahiatal hernia. No such cases have been reported in the literature to date. In addition, ICG fluorescence imaging was used to assess blood flow during the gastric resection.

### Diagnostic challenges and CT differentiation

One point to emphasize regarding adult diaphragmatic hernias is the high risk of misdiagnosis.^28)^ Both lung abscess and incarcerated diaphragmatic hernia can appear on CT as intrathoracic cavitary lesions with an air–fluid level, which may lead to diagnostic confusion. In particular, when a lung abscess is located at the lung base, the location of the air–fluid level may closely resemble that seen in a diaphragmatic hernia as shown in **Fig. 1A**, making differentiation challenging.

However, several imaging findings favor the diagnosis of diaphragmatic hernia rather than lung abscess. Characteristic CT findings of incarcerated diaphragmatic hernia and lung abscess are summarized in **Table 2**. First, continuity between the intrathoracic lesion and intra-abdominal organs, such as the stomach or intestine, is a key diagnostic feature of diaphragmatic hernia, as shown in **Fig. 1B**. Second, the enhancement pattern of the lesion wall on contrast-enhanced CT is helpful for differentiation. In lung abscesses, the abscess wall typically demonstrates contrast enhancement due to inflammatory hypervascularity. In contrast, in diaphragmatic hernia with incarcerated gastrointestinal organs, as shown in **Fig. 1A** and **1B**, poor contrast enhancement of the stomach or intestinal wall reflects ischemia and strongly suggests herniation rather than an infectious pulmonary process. The condition was initially diagnosed as a left lung abscess at another hospital, delaying definitive treatment by 1 day and potentially contributing to gastric necrosis. Although diaphragmatic hernia incarcerations and perforations are relatively uncommon, they are life-threatening. The mortality rate for gastric perforation in the thoracic cavity ranges from 42% to 56%.^29,30)^ Given these dangers, parahiatal hernia should remain on the differential diagnosis list whenever a cavitary lesion is seen in the lung, even though it is a rare condition.

**Table 2:** Characteristic CT findings of incarcerated diaphragmatic hernia and lung abscess

CT findings	Air-fluid level	Continuity with abdominal organs	Wall contrast enhancement
Incarcerated diaphragmatic hernia	Present in the thoracic cavity	Present	Poor or absent
Lung abscess	Present in the thoracic cavity	Absent	Marked

### Surgical considerations

The optimal surgical strategy for parahiatal hernia remains controversial due to its rarity. There are several surgical considerations, including whether to place a mesh, whether to perform fundoplication, and whether to use ICG fluorescence imaging during resection of the incarcerated organ. There have been no long-term studies investigating the recurrence of parahiatal hernia, and the efficacy of mesh placement for parahiatal hernia remains unclear.^21)^ Fundoplication was performed in several reported cases (**Table 1**), but its efficacy is also uncertain. Mesh placement was avoided due to the risk of mesh infection and prolonged operation time. The necrotic tissue was present, and gastric resection was required, both of which were considered potential risk factors for mesh infection. In addition, as the surgery was performed as an emergency procedure, it was necessary to minimize operative time considering the patient’s overall condition. Fundoplication was also not performed for the same reason, but the patient did not develop any symptoms of gastroesophageal reflux. ICG fluorescence imaging played a crucial role in intraoperative decision-making. The gastric fundus, which is anatomically adjacent to the cardia, most frequently herniates (**Table 1**). When incarceration occurs, necrosis may extend to the cardia or partial resection may be deemed likely to cause a stenosis; in such cases, proximal gastrectomy is required and results in the loss of cardial function. Therefore, precise identification of the minimum yet sufficient extent of gastric resection is essential. Determining the extent of viable gastric tissue based solely on visual inspection can be unreliable, particularly in cases of borderline ischemia. ICG enables real-time assessment of tissue perfusion. On visual inspection, necrosis was mainly confined to the gastric fundus; however, discoloration extended close to the cardia, suggesting that proximal gastrectomy might have been necessary. The use of ICG fluorescence imaging allowed accurate assessment of gastric perfusion, enabling clear determination of the resection margin and avoidance of excessive gastric resection. However, several limitations should be considered when applying it. First, its limited tissue penetration depth is an important constraint. It is generally reported to be approximately 5–10 mm,^31)^ whereas the thickness of the gastric wall is typically around 3–4 mm.^32)^ However, gastric wall thickness may vary due to various factors like inflammatory edema, and therefore mucosal ischemia may not always be accurately reflected by the fluorescence signal.^31)^ In addition, fluorescence intensity can be influenced by technical factors such as the distance between the tissue and the light source, as well as ambient lighting conditions. Furthermore, determining the boundary line based on ICG fluorescence imaging inevitably involves subjective interpretation by the surgeon.^33)^ Therefore, these limitations should be carefully considered when applying this technique.

### Clinical implications

This case emphasizes that parahiatal hernia, although rare, can progress to life-threatening gastric necrosis when incarceration occurs. Accurate interpretation of CT findings is critical for early diagnosis. Furthermore, laparoscopic repair and organ resection combined with perfusion assessment using ICG fluorescence imaging may allow safe, minimally invasive, and organ-preserving management even in emergency situations. Accumulation of further cases is necessary to clarify optimal repair strategies and long-term outcomes.

## CONCLUSIONS

We report an extremely rare case of primary parahiatal hernia complicated by gastric necrosis. Although uncommon, parahiatal hernias can be life-threatening when associated with perforation. Therefore, this condition should be considered in the differential diagnosis of cavitary lung lesions.
